# Genome Structure of the Opportunistic Pathogen *Paracoccus yeei* (*Alphaproteobacteria*) and Identification of Putative Virulence Factors

**DOI:** 10.3389/fmicb.2018.02553

**Published:** 2018-10-25

**Authors:** Robert Lasek, Magdalena Szuplewska, Monika Mitura, Przemysław Decewicz, Cora Chmielowska, Aleksandra Pawłot, Dorota Sentkowska, Jakub Czarnecki, Dariusz Bartosik

**Affiliations:** Department of Bacterial Genetics, Faculty of Biology, University of Warsaw, Warsaw, Poland

**Keywords:** *Paracoccus yeei*, opportunistic pathogen, virulence factors, mobilome, chromids, plasmids, genomic islands, evolution of pathogenic bacteria

## Abstract

Bacteria of the genus *Paracoccus* are common components of the microbiomes of many naturally- and anthropogenically shaped environments. One species, *Paracoccus yeei*, is unique within the genus because it is associated with opportunistic human infections. Therefore, strains of *P. yeei* may serve as an interesting model to study the transition from a saprophytic to a pathogenic lifestyle in environmental bacteria. Unfortunately, knowledge concerning the biology, genetics and genomic content of *P. yeei* is fragmentary; also the mechanisms of pathogenicity of this bacterium remain unclear. In this study we provide the first insight into the genome composition and metabolic potential of a clinical isolate, *P. yeei* CCUG 32053. This strain has a multipartite genome (4,632,079 bp) composed of a circular chromosome plus eight extrachromosomal replicons pYEE1–8: 3 chromids and 5 plasmids, with a total size of 1,247,173 bp. The genome has been significantly shaped by the acquisition of genomic islands, prophages (*Myoviridae* and *Siphoviridae* phage families) and numerous insertion sequences (ISs) representing seven IS families. Detailed comparative analysis with other complete genomic sequences of *Paracoccus* spp. (including *P. yeei* FDAARGOS_252 and TT13, as well as non-pathogenic strains of other species in this genus) enabled us to identify *P. yeei* species-specific genes and to predict putative determinants of virulence. This is the first attempt to identify pathoadaptive genetic information of *P. yeei* and to estimate the role of the mobilome in the evolution of pathogenicity in this species.

## Introduction

The genus *Paracoccus* (*Alphaproteobacteria*) contains several hundred strains classified into over 50 species. These bacteria are important components of the microbiomes of different pristine and polluted environments. Many *Paracoccus* strains have been isolated from soil, brines and marine sediments, sewage, and biofilters (e.g., Urakami et al., 1990; Siller et al., 1996; Lipski et al., 1998; Tsubokura et al., 1999; Lee et al., 2004; Liu et al., 2006; Balouiri et al., 2016). Some strains were also identified in association with plant rhizospheres or with other organisms, including ticks, marine bryozoans and corals (Pukall et al., 2003; Machado-Ferreira et al., 2012; Carlos et al., 2017). Interestingly, one species, *Paracoccus yeei*, has been implicated in opportunistic infections of humans, which is unique within the genus (Daneshvar et al., 2003). Therefore, strains of *P. yeei* represent a very interesting model to study the molecular bases of the transition from a saprophytic to a pathogenic lifestyle.

*Paracoccus yeei* strains were originally classified as members of the Centers for Disease Control (CDC) group 2 of eugonic oxidizers (EO-2), which comprises Gram-negative bacterial strains of unknown taxonomic position, isolated from a variety of human sources in diverse geographic locations. This heterogeneous set of strains is further divided into several sub-groups on the basis of differences in their fatty acid profiles, cellular morphology and pigment production (Hudson et al., 1987). This permitted the establishment of a group of strains originating from various clinical sources in the United States and Canada, which are genetically related to the genus *Paracoccus* but distinct from other *Paracoccus* species. Molecular studies revealed that these strains represent a novel species for which the name *P. yeei* was proposed (Daneshvar et al., 2003).

There are currently fifty-three 16S rDNA sequences from *P. yeei* strains deposited in the NCBI database. Despite this relatively low number, strains of this species seem to be widely distributed, having been isolated from different environments in North and South America, Europe, Asia, and Africa. So far, there have been only 19 cases of infection with *P. yeei* documented in the literature. However, this is likely to be a considerable underestimate because current diagnostic tests do not detect this bacterium (Sack et al., 2017). Nothing is known about the mechanisms of pathogenicity of *P. yeei* or the basis of its interaction with eukaryotic cells. Moreover, the identity of any reservoir and the transmission route of this bacterium remain unclear. It is noteworthy that the isolates causing clinical human infections are not associated with a specific disease unit. The type strain *P. yeei* ATCC BAA-599 was isolated from the dialysate of a patient with peritonitis (Daneshvar et al., 2003). Other strains have been recovered from a variety of clinical conditions, e.g., myocarditis in a transplanted heart (Schweiger et al., 2011), corneal transplantation (Kanis et al., 2010), bacteremia (Sack et al., 2017), keratitis (Courjaret et al., 2014), otitis (Daneshvar et al., 2003), and dermatologic lesions (Funke et al., 2004).

It is likely that the unique lifestyle of pathogenic *P. yeei* strains results from the acquisition of foreign DNA of adaptive value via horizontal gene transfer (HGT). The major agents of HGT are mobile genetic elements (MGEs), such as widely distributed plasmids and transposable elements (TEs). In previous studies it was demonstrated that MGEs are common in *Paracoccus* spp. (e.g., Dziewit et al., 2012; Maj et al., 2013; Czarnecki et al., 2017). In several analyzed strains numerous functional TEs were identified, including ISs, composite and non-composite transposons, transposable modules (TMos) and a large transposable genomic island Tn*Ppa1*. Genomic analysis of two methylotrophic strains, *P. aminophilus* JCM 7686 and *P. aminovorans* JCM 7685, showed that bacteria of this genus may have multipartite genomes (Dziewit et al., 2015a; Czarnecki et al., 2017) comprising numerous extrachromosomal replicons (ECRs; chromids and plasmids) with diverse structures and properties. Some of the plasmids of *P. aminophilus* and *P. aminovorans* were shown to be important lifestyle-determining elements, carrying genetic information crucial for colonization of the primary ecological niche of their host strains (soil contaminated with dimethylformamide) (Dziewit et al., 2010; Czarnecki et al., 2017). Analogous MGEs may also determine the pathogenic properties of *P. yeei* strains.

This encouraged us to perform a genomic analysis of *P. yeei* CCUG 32053, a strain isolated in the United States in 1981 from a patient with an eye infection. The aim was to reveal the structure and composition of the CCUG 32053 genome, paying special attention to its mobile DNA. When this project was initiated little information was available concerning the genome structure or MGEs of *P. yeei*. The only relevant accession in the GenBank database was a partial genomic sequence of the aforementioned type strain ATCC BAA-599 (70 scaffolds and 87 contigs, without further assignment of chromosomal or extrachromosomal origin). Recently (in March and November 2017) the complete genomic sequences of two other strains of *P. yeei*, FDAARGOS_252 and TT13 (Lim et al., 2018), isolated from urine suprapubic aspirate and human skin, respectively, were released (acc. nos. NZ_CP020440–47 and NZ_CP024422–28). These data revealed the presence of numerous large ECRs in *P. yeei* (7 in FDAARGOS_252 and 6 in TT13) with a total size of 1,207,416 and 1,032,138 bp, respectively.

In this report we present a detailed comparative genomic analysis of *P. yeei* strains CCUG 32053, FDAARGOS_252 and TT13 in relation to the genomes of other *Paracoccus* species. We also describe the first attempt to identify pathoadaptive genetic information of *P. yeei* and to estimate the role of mobile DNA in the evolution of pathogenicity in this species.

## Materials and Methods

### Strains and Culture Conditions

Strain *P. yeei* CCUG 32053, subjected to genomic analysis in this study, was purchased from the Culture Collection of the University of Gothenburg (CCUG) (Sweden).

*Paracoccus yeei* CCUG 32053R, a rifampicin-resistant derivative of the wild-type CCUG 32053 strain (Dziewit et al., 2012) and *Escherichia coli* TG1 (Sambrook and Russell, 2001) were used as plasmid recipients. *E. coli* DH5α (Hanahan, 1983) was the host strain of helper plasmid pRK2013. The following strains were used for the analysis of plasmid host range: (i) *Alphaproteobacteria* – *P. solventivorans* DSM 11592R (Bartosik et al., 2003b), *P. versutus* UW1R (Bartosik et al., 2002), *Agrobacterium tumefaciens* LBA 288R (Hooykaas et al., 1980), *Rhizobium etli* CE3 (Noel et al., 1984), (ii) *Betaproteobacteria* – *Alcaligenes* sp. LM16R (Dziewit et al., 2015b) and (iii) *Gammaproteobacteria* – *Pseudomonas* sp. LM7R, *Acinetobacter* sp. LM3R, *Psychrobacter* sp. LM26R and *Stenotrophomonas* sp. LM24R (Dziewit et al., 2015b).

All strains were grown in lysogeny broth (LB) medium (Sambrook and Russell, 2001) at 37°C (*E. coli*) or 30°C (other strains). Where necessary, the medium was supplemented with antibiotics at the following concentrations: kanamycin, 50 μg/ml; rifampicin, 50 μg/ml; tetracycline, 1 μg/ml for *P. yeei* and 20 μg/ml for *E. coli* strains.

### Physiological Analyses of *P. yeei* CCUG 32053

The temperature, pH and salinity tolerance of *P. yeei* CCUG 32053 were analyzed by monitoring changes in the optical density (OD) of cultures (in comparison with non-inoculated controls) according to previously described procedures (Dziewit et al., 2013). Motility was tested on soft LB agar plates containing 0.3, 0.35, or 0.4% (w/v) agar. The plates were inoculated with bacteria using a sterile toothpick and incubated at 30°C for 48 h. The minimum inhibitory concentrations (MICs) of selected antibiotics were established as previously described (Balouiri et al., 2016).

### DNA Isolation, Standard Genetic Manipulations and PCR Conditions

Plasmid DNA was isolated using the alkaline lysis procedure (Birnboim and Doly, 1979) and when required, the DNA was further purified by CsCl-ethidium bromide gradient centrifugation (Sambrook and Russell, 2001). The visualization of mega-sized replicons was achieved by in-gel lysis and DNA electrophoresis (Wheatcroft et al., 1990). DNA manipulation procedures were performed using standard methods (Sambrook and Russell, 2001). All plasmids constructed and used in this study are described in Supplementary Table S1.

DNA amplification by PCR was performed in a Mastercycler (Eppendorf) using synthetic oligonucleotides (listed in Supplementary Table S1), Phusion polymerase (Thermo Fisher Scientific), dNTPs and appropriate template DNAs, as described previously (Bartosik et al., 2003a).

### Introduction of Plasmid DNA Into Bacterial Cells

Chemical transformation of *E. coli* cells was performed by a standard method (Kushner, 1978). Plasmid DNA was introduced into *Paracoccus* spp. strains by triparental mating using helper *E. coli* strain DH5α carrying plasmid pRK2013 (containing the transfer system of plasmid RP4) (Ditta et al., 1980). Briefly, overnight cultures of the donor (*E. coli* TG1 carrying the appropriate mobilizable plasmid), recipient and helper strains were harvested by centrifugation and the cells washed twice in fresh medium lacking antibiotics. The three cell suspensions were then combined in a ratio of 1(D):2(R):1(H) and 100 μl of the mixture was plated on LB agar medium. After overnight incubation, the bacteria were washed off the plate and suitable dilutions were plated on media containing the appropriate antibiotics to select the transconjugants.

### Identification of Functional Transposable Elements

Trap plasmid pMEC1 (Km^r^) (Bartosik et al., 2003a) was used for the identification of functional TEs of *P. yeei*. This plasmid contains the *cI-tetA* cassette, composed of (i) a silent tetracycline resistance gene *tetA* under the control of the bacteriophage lambda pR promoter, and (ii) the gene encoding the lambda cI repressor. Inactivation of the repressor gene (e.g., through insertion of an IS), results in constitutive expression of tetracycline resistance (Schneider et al., 2000). The plasmid was transferred from *E. coli* TG1 to *P. yeei* CCUG 32053R by triparental mating. An overnight culture of a kanamycin and rifampicin-resistant transconjugant was then plated on LB agar medium supplemented with tetracycline. Of the resulting tetracycline-resistant clones, 150 were analyzed for the presence of pMEC1 derivatives containing integrated TEs. The TE insertions were localized by performing PCR amplifications with isolated plasmid DNA as the template and sets of cassette-specific primers (Supplementary Table S1) (Bartosik et al., 2003a). All trapped TEs were sequenced and their sequences compared with the ISfinder database (Siguier et al., 2006).

### Genome Sequencing

The *P. yeei* CCUG 32053 genome was sequenced by combining the Oxford Nanopore and Illumina technologies. Whole genome sequencing was performed using a MinION instrument with a R9.4 flow cell and 1D ligation kit SQK-LSK108, and an Illumina MiSeq instrument with 2×300 paired-end mode and v3 MiSeq chemistry kit, which produced 77,644 (257 Mb) and 1,639,864 (488.5 Mb) reads, respectively. Raw MinION data base calling was performed using Albacore v2.0.2 (Oxford Nanopore Technologies). Adaptors were removed using Porechop v.0.2.1^1^. Genome assembly was carried out using Canu v1.6 (Koren et al., 2017) and subsequently polished with Racon v0.5.0 (Vaser et al., 2017). Illumina reads were then mapped against the acquired genome using bwa v0.7.15-r1142-dirty (Li and Durbin, 2010) and final sequence correction was performed using Pilon v1.21 (Walker et al., 2014). The genome assembly was verified by comparison with assemblies obtained using Newbler De Novo Assembler v3.0 (454 Sequencing System Software, Roche) and SPAdes v3.11.1 (Bankevich et al., 2012).

### Bioinformatic Analyses

Automatic annotation was performed using RAST on the PATRIC 3.4.13 platform (Wattam et al., 2017). After automatic annotation, the sequence information was refined manually in Artemis following homology searches conducted with BLASTp and BLASTn tools via the National Center for Biotechnology Information (NCBI) website^2^ using default settings (Altschul et al., 1997). Putative tRNA genes were identified using tRNAScan-SE (Lowe and Eddy, 1997). rRNA operons were identified by comparison with rRNA genes from other *Paracoccus* spp. using BLASTn.

Clusters of orthologous groups (COGs) categories were assigned for each protein using a local RPS-BLAST search against the COG database (last modified January 22, 2015). An *e*-value threshold of 1*e*-30 was applied so that only the best BLAST hits were considered (Tatusov et al., 2003).

Relaxases (MOB) were classified according to sequence similarity (Garcillan-Barcia et al., 2009). Toxin-antitoxin modules were identified using TAfinder (Shao et al., 2011). TEs were defined using ISSaga and the ISfinder website (Siguier et al., 2006; Varani et al., 2011), and then manually curated. Comparison searches for ISs were performed with ISfinder. Prophages were predicted with the PhiSpy algorithm (Akhter et al., 2012). Phage families were predicted based on analyses of the head-neck-tail module genes conducted at the Virfam server (Lopes et al., 2014).

The core genome of the genus *Paracoccus* was defined based on the complete genomes of *P. yeei* CCUGG 32053 and six other strains, i.e., *P. aminophilus* JCM 7686 (Dziewit et al., 2014), *P. aminovorans* JCM 7685 (Czarnecki et al., 2017), *P. contaminans* RKI16-01929^T^ (Aurass et al., 2017), *P. denitrificans* PD1222 (NC_008686–8), *P. yeei* FDAARGOS_252 (NZ_CP020440–47) and *P. yeei* TT13 (Lim et al., 2018). Proteins encoded within the genomes were used in all against all BLASTp searches using the following thresholds: *e*-value 1*e*-30, 70% identity and 85% query coverage of the high scoring pair (HSP). In addition, all observed sequence similarities were filtered and only reciprocated homologies above the threshold were retained. Based on this analysis, all proteins were clustered into groups reflecting reciprocated similarity and the composition of these groups was checked to see if they contained proteins from all seven genomes (core proteins), proteins from a single genome (singletons), or proteins common to the three *P. yeei* species only (*P. yeei* species-core proteins). A similar approach with a different manner of grouping was applied to identify species- and replicon-specific gene clusters.

*In silico* metabolism reconstruction was based on information from the KEGG database (Kanehisa et al., 2017). The subcellular localization of the putative proteins was predicted with the use of PSORTb 3.0.2 server (Yu et al., 2010). The presence of signal peptides in the amino acid sequences of putative proteins was predicted using SignalP Server 4.1 with default settings (Petersen et al., 2011). Proteins secreted via non-classical signal peptide-independent secretion pathways were predicted using the SecretomeP 2.0 Server (Bendtsen et al., 2005). The statistical cut off was the default setting for both SignalP 4.1 and the SecretomeP 2.0 servers.

Protein datasets from the Virulence Factor Database (Chen et al., 2016) and MvirDB (Zhou et al., 2007) were used for BLASTp searches (*e*-value threshold 1*e*-30) against the proteomes of *P. yeei* CCUG 32053 and six other *Paracoccus* spp. strains to identify putative virulence determinants. The results were manually curated.

EasyFig (Sullivan et al., 2011) and Circoletto (Darzentas, 2010) were used to perform comparative genomic analyses and visualize the results. A detailed description of the use of this software is given in Supplementary Table S2.

Phylogenetic analysis of the genus *Paracoccus* was based on alignment of concatenated nucleotide sequences of selected core genes: *atpD*, *dnaA*, *dnaK*, *gyrB*, *recA*, *rpoB*, and *thrC* (homologous genes of *Rhodobacter denitrificans* OCh 114 and *Roseobacter sphaeroides* ATCC 17025 were used as an outgroup). The nucleotide alignments were obtained with translatorX v.1.1 (Abascal et al., 2010) and MUSCLE v.3.8.31 (Edgar, 2004) as amino acid sequences aligner. Alignments for each gene were curated with trimAl v1.2rev57 (Capella-Gutierrez et al., 2009) using -gappyout option to remove poorly aligned regions. Then, concatenated genes (with 13779 variable sites) were analyzed with PartitionFinder 2 checking all models with AICC score selection method and greedy search scheme (Lanfear et al., 2012, 2017) for selecting best-fit partitioning scheme and model of evolution. Based on that, GTRGAMMAI substitution model was applied in RAxML v8.2.12 (Stamatakis, 2014) with 2000 regular bootstrap replicates performed on the best Maximum Likelihood (ML) tree selected from 100 independently generated ML starting trees.

### Nucleotide Sequence Accession Numbers

The nucleotide sequences of the *P. yeei* CCUG 32053 chromosome and extrachromosomal replicons pYEE1, pYEE2, pYEE3, pYEE4, pYEE5, pYEE6, pYEE7, and pYEE8 were deposited in GenBank (NCBI) with the respective accession numbers CP031078, CP031079, CP031080, CP031081, CP031082, CP031083, CP031084, CP031085, and CP031086. The nucleotide sequences of two newly identified ISs (IS*Pye1*–IS*Pye68*) were deposited in the ISfinder database (Siguier et al., 2006). For the sake of brevity, the locus names of protein-encoding genes were shortened throughout this report (e.g., PY_00001 instead of PY32053_00001).

## Results

### Physiological Characterization of *P. yeei* CCUG 32053

Preliminary physiological characterization of *P. yeei* CCUG 32053 revealed that it can grow at temperatures ranging from 15 to 37°C, which is typical for mesophilic bacteria. The strain grew in LB medium at pH values of 5–9 and it could tolerate NaCl at a concentration of 6% (w/v). Further analyses revealed that CCUG 32053 is (i) a non-motile, (ii) non-hemolytic, (iii) denitrifying bacterium, and (iv) unable to form biofilms on plastic surfaces (micro-well plates) (data not shown). The MICs for several antibiotics (representing aminoglycosides, β-lactam antibiotics, second-generation fluoroquinolones and macrolides) did not reveal any unequivocal resistance phenotype (Supplementary Table S3).

### Genome Description

The complete nucleotide sequence of *P. yeei* CCUG 32053 was determined and assembled as described in Section “Materials and Methods.” The average G + C content of the sequence is 64.8%, which falls within the range found for the other fully sequenced *Paracoccus* spp. genomes (63.4–68.7% G + C). The CCUG 32053 strain has a composite genome consisting of a circular chromosome (3,421,679 bp) and eight extrachromosomal replicons (ECRs) named pYEE1 to pYEE8 (Table 1), whose presence was confirmed by electrophoretic methods (Supplementary Figure S1). The eight ECRs range in size from 8143 bp (pYEE8) to 482,273 bp (pYEE1) (Table 1). Their combined size is 1,247,173 bp, which represents 26.7% of the entire genome.

**Table 1:** General features of the *Paracoccus yeei* CCUG 32053 genome.

General features	Chromosome	pYEE1	pYEE2	pYEE3	pYEE4	pYEE5	pYEE6	pYEE7	pYEE8
REP type	*dnaA*	*repB1*	*dnaA-like*	*repABC*	*repB2*	*repC*	Rep_3	HTH_36	HTH_36
Size (bp)	3 421 679	482 273	296 393	201 841	156 389	74 143	18 339	9 652	8 143
G + C content (%)	67.58%	67.98%	68.43%	64.65%	68.06%	61.24%	61.44%	55.79%	64.58%
Coding percentage	90.04%	88.7%	91.7%	78.8%	91.3%	89.04%	76.3%	81.9%	63.0%
CDSs	3 378	418	275	198	139	80	21	15	9
Pseudogenes	80	23	7	28	8	7	2	–	2
tRNA genes	50	1	–	1	1	–	–	–	–
16S-23S-5S rRNA operons	3	–	–	–	–	–	–	–	–
Core genes*^a^*	871	4	14	4	7	0	0	0	0
Species-specific genes*^b^*	119	79	45	4	15	3	1	0	0
Strain-specific genes	148	47	29	35	12	6	4	2	3
Complete prophages	3	–	–	–	–	–	–	–	–
GTA regions	1	–	–	–	–	–	–	–	–
Transposase genes (incl. truncated)	58	15	3	29	7	7	3	1	1
Complete ISs	37	7	2	13	6	5	3	1	–

*^a^conserved in all complete genomes of Paracoccus spp. ^b^conserved in all complete genomes of P. yeei and not present in other Paracoccus spp. Genomes.*

The CCUG 32053 chromosome exhibits a highly polarized nucleotide composition indicating two replichores, and this strand asymmetry is demonstrated by plotting a GC skew graph (Supplementary Figure S1). The GC skew splits this DNA molecule into two regions, with shift points correlated with the origin (*oriC*) and terminus (*ter*) of replication. The predicted *oriC* site (2963373–2963778) is located within an AT-rich (58.4%) intergenic sequence, in the vicinity of *parAB* genes, which are involved in chromosome segregation in other bacteria (Reyes-Lamothe et al., 2012). This site is composed of three different putative DnaA-binding boxes, matching the consensus sequence TTWTNCACA (W – A or T; N – any nucleotide) (Schaper and Messer, 1995). Interestingly, the *dnaA* gene, encoding the chromosome replication initiator protein, is at a distance of 964,841 bp from the *oriC*. An analogous *oriC* organization and distant localization of the *dnaA* gene is also observed in the chromosomes of the *P. yeei* strains FDAAARGOS_252 and TT13.

Gene annotation revealed that the CCUG 32053 genome contains 4533 protein-coding sequences (CDSs): 3378 located within the chromosome (75%) and 1115 within the ECRs (25%) (Table 1). These CDSs cover 89.4% of the entire CCUG 32053 genome. In addition, 3 chromosomally encoded sets of rRNA operons, 50 tRNA genes and one tmRNA gene were identified. tRNAs for all 20 amino acids and tRNA for selenocysteine are encoded by the chromosome. Three additional tRNA genes (two for proline and one for leucine) are located in three different ECRs – pYEE1, pYEE3, and pYEE4, respectively (Table 1). The anticodons of the pYEE1- and pYEE4-encoded tRNAs are unique in the CCUG 32053 genome, while the pYEE3-encoded tRNA has the same anticodon as the two chromosomally encoded proline tRNAs.

### Extrachromosomal Replicons

To shed light on the origin of the CCUG 32053 ECRs, their conserved backbones, comprised of replication (REP), stabilization and transfer systems were identified and characterized. This analysis showed that all predicted REPs are highly conserved in the genomes of other strains of *P. yeei*. All of them are also widely distributed among diverse ECRs of *Alphaproteobacteria* (Cevallos et al., 2008; Petersen et al., 2013).

To test the host range of the plasmids, their REP regions were cloned in mobilizable shuttle plasmids and introduced by triparental mating into rifampicin-resistant strains representing different classes of *Proteobacteria* (listed in section “Materials and Methods”). This analysis revealed that all the REPs are functional in *Alphaproteobacteria*, but not in *Beta*- or *Gammaproteobacteria*, which points to their relatively narrow host range.

Two ECRs of the studied strain, pYEE1 (the largest ECR of CCUG 32053) and pYEE4 (Table 1), encode related but distinct RepB-type replication initiator proteins (designated RepB1 and RepB2, respectively) sharing 28% amino acid sequence identity. Another large replicon, pYEE2, encodes a DnaA-like replication initiator characteristic of mega-sized plasmids occurring in bacteria of the *Roseobacter* clade (Petersen et al., 2013). Related REP modules provide replication functions to the chromids of *P. denitrificans* PD1222 (chromosome 2), *P. aminophilus* JCM 7685 (pAMI5) (Dziewit et al., 2014) and *P. aminovorans* JCM 7686 (pAMV3) (Czarnecki et al., 2017). In the immediate vicinity of the predicted REPs of the aforementioned ECRs, there are two genes (*parA* and *parB*), comprising a putative partitioning system (PAR) responsible for the active segregation of plasmid molecules into daughter cells at cell division. Two other ECRs, pYEE3, and pYEE5, encode RepC replication initiators. The *repC* gene of pYEE3 and the partitioning *repA* and *repB* genes form a putative *repABC* operon, while *repC* of pYEE5 is a solo gene not associated with a PAR module. The 3 smallest CCUG 32053 replicons, pYEE6, pYEE7, and pYEE8, encode distinct Rep_3 or HTH_36 family proteins that are typical for diverse small ECRs. The smallest ECR, pYEE8, shares significant sequence similarity with plasmid pMOS6 of an astaxanthin producing strain *P. marcusii* OS22 (Maj et al., 2013). These two plasmids have highly similar backbones, although they contain different REP regions (data not shown).

A common feature of all the ECRs of CCUG 32053 is the presence of toxin-antitoxin (TA) systems, which confer plasmid stabilization by eliminating plasmid-less cells at the post-segregational level (Diaz-Orejas et al., 2017). The TA *loci* encode two elements: (i) a toxin protein that binds a specific cellular target and (ii) an antitoxin (protein or antisense RNA), which counteracts the toxin. All of the pYEE *loci* are type II TA systems, composed of toxin and antitoxin proteins that are encoded in a single operon (Diaz-Orejas et al., 2017).

One of the identified *loci* belongs to the *parDE* superfamily of TA systems (pYEE6). Six *loci* encode toxins from the RelE/ParE superfamily (pYEE8, two *loci* of pYEE7, pYEE5, pYEE4, pYEE3). Another four encode toxins related to VapC (with pilT N-terminal domain) (pYEE6, two *loci* of pYEE4, pYEE2). However, these proteins are paired with antitoxins typically associated with toxins of other TA families (PhD/YefM, CopG, and MazE) or are of unknown origin. This is consistent with the occurrence of antitoxin shuffling between different TA systems resulting in the generation of hybrid addiction modules (Lee and Lee, 2016).

None of the aforementioned shuttle plasmids, containing pYEE REP modules, could be introduced into strain CCUG 32053 to replace the parental replicon (data not shown). This points to the activity of the TA modules, which precludes removal of their carrier replicons.

The CCUG 32053 ECRs do not carry clusters of conjugal transfer genes, although four of them encode predicted relaxases – i.e., proteins playing a key role in conjugative mobilization (MOB modules) (Wawrzyniak et al., 2017) – representing the MOB_Q_ (pYEE1, pYEE2, and pYEE3) and MOB_HEN_ (pYEE6) families (Garcillan-Barcia et al., 2009).

### Transposable Elements

ISSaga was used to scan the genome sequence of CCUG 32053 for the presence of TEs. This approach revealed that this strain contains numerous ISs. Both partial and complete ISs were identified, including full length elements disrupted by the transposition of other ISs, which can be reconstructed *in silico* (Supplementary Table S4). To get a broader view on the diversity of the transposable mobilome of this species, the analysis was expanded to include other strains of *P. yeei* (FDAARGOS_252 and TT13). Interestingly, as many as 67 novel ISs (IS*Pye1*–IS*Pye67*) were identified within the three complete *P. yeei* genome sequences, with only one previously known element – an isoform of IS*Ppa9* in FDAARGOS_252 – originally detected in *P. pantotrophus* DSM 65 (Dziewit et al., 2012). Notably, only one complete element was common to all three strains (IS*Pye9* of the IS*6* family), which indicates huge interspecies diversity of the *P. yeei* TEs.

**FIGURE 1:**
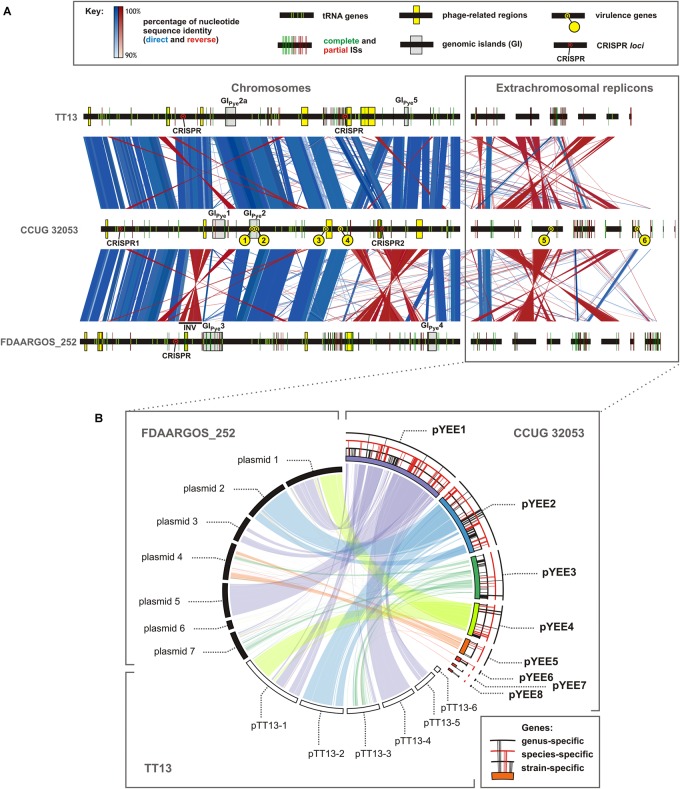
Comparative genomic analysis of three strains of *Paracoccus yeei*: TT13, CCUG 32053, and FDAARGOS_252. **(A)** Whole genome synteny analysis, visualized using EasyFig software. The localization of the identified tRNA genes, insertion sequences, phage-related regions, genomic islands, CRISPR *loci* and the putative CCUG 32053 virulence-related genes is presented as indicated in the key. INV - inverted a 217-kb-long segment of chromosomal DNA (in FDAAGOS_252) flanked by two copies of IS*Pye28*. **(B)** Relationship between ECRs of CCUG 32053 and ECRs of other strains of *P. yeei*, visualized using Circoletto software. The localization of the genus-, species- and strain-specific genes within the replicons of the CCUG 32053 strain is presented as indicated in the key. The detailed description of the methodology used to generate the figure is to be found in Supplementary Table S2.

Our analysis revealed that ISs constitute approximately 3% of the CCUG 32053 genome. This strain carries 74 complete (representing 32 different elements) and 54 partial IS sequences (Supplementary Table S5). Based on comparative analysis, the elements were assigned to 7 IS families: IS*3* (groups IS*3*, IS*51*, IS*150*, IS*407*), IS*5* (groups IS*5*, IS*427*, IS*903*), IS*30*, IS*66*, IS*256*, IS*110*, and IS*1182*. The most prevalent elements were those of the IS*5* (24 complete elements) and IS*66* (15) families, and the most dynamic in transposition were two elements, IS*Pye41* (IS5 family/IS*903* group) and IS*Pye42* (IS*3* family/IS*51* group), that are represented by 12 and 9 copies, respectively. The distribution of the identified ISs in the genome is uneven, with the majority (both complete and partial) residing within three replicons: the chromosome (59), pYEE3 (31) and pYEE1 (15) (Figure 1A).

The genomes of *P. yeei* strains FDAARGOS_252 and TT13 carry a total of 204 complete ISs (and 152 partial elements), whose genomic distribution is also presented in Figure 1A. These TEs are more diverse, since they represent 15 different IS families (IS*1595*, IS*4*, ISL*3*, IS*630*, IS*481*, IS*701*, IS*21*, and IS*6*, plus those identified in CCUG 32053).

To verify the transposition activity of the ISs of CCUG 32053 identified *in silico*, a positive selection trapping strategy using plasmid pMEC1 was employed (Bartosik et al., 2003a). Transposition events occurring within the pMEC1 cassette were selected on LB agar plates supplemented with tetracycline (selection of tetracycline-resistant clones; see section “Materials and Methods” for details). Analysis of 150 tetracycline resistant clones revealed that around 40% of them carried pMEC1-derivatives containing inserts with sizes of approximately 1.4 kb (50 clones) and 1 kb (11 clones). DNA sequencing revealed that this *in vivo* approach led to the capture of only two elements: (i) IS*Pye38* (1366 bp) of the IS*5* family, and (ii) IS*Pye41* (1055 bp) of the IS*5* family (IS*903* group).

### Prophages and CRISPR *Loci*

The CCUG 32053 genome contains 5 phage-related regions, all located within the chromosome (Figure 1A). Three of them are complete genomes of the *Myoviridae* and *Siphoviridae* phage families. The *Myoviridae*-family prophage (723645–773805) is located in the immediate vicinity of two tRNA-Pro(TGG) genes. Analysis of the surrounding DNA revealed the presence of two identical 52-nt-long direct repeats located at the start of these tRNA genes. This could represent the potential attachment site of the phage whose integration resulted in duplication of the tRNA-Pro(TGG) gene.

The two other prophages originate from different members of the *Siphoviridae* family. For one of them (1583112–1633328) no potential attachment site was identified. Nonetheless, its borders could be readily distinguished, with the prophage integrated between highly conserved genes of the tRNA modification operon (PY_01612–01616 and PY_01684). The genome of the second phage (2976489–2995275) is integrated within the tRNA-SeC(p) gene, as judged from 15-nt-long DRs (identical to a terminal part of this gene) bordering the prophage.

The two remaining phage-related regions contain an incomplete prophage and a putative operon encoding gene transfer agents (GTA), i.e., phage-like entities that mediate HGT (Lang et al., 2012). The prophage remnant (1217673–1236476) only contains an integrase gene, potential replication initiation genes and a disrupted terminase gene. The GTA operon (2046777–2060272) is composed of 17 ORFs. It is highly similar (at the DNA sequence level) to GTA-encoding regions of *P. yeei* strains FDAARGOS 252 and TT13 (99% identity), *P. aminovorans* JCM 7685 and *P. denitrificans* PD1222 (82% identity) and *P. aminophilus* JCM 7686 (76% identity), with preserved synteny and at least 95% sequence coverage.

The strain CCUG 32053 also carries a putative CRISPR-based anti-phage defense system. The chromosome of this strain contains two distantly located CRISPR *loci* (CRISPR1, CRISPR2), separated by 921 kb. However, only one of them (CRISPR1; 2181604–2184133) is accompanied by a cluster of CRISPR-associated *cas*-*cse* genes. These genes encode eight putative proteins, including Cas1, Cas2, Cas3, Cse1, and Cse4 (PY_02240–PY_02247), which are characteristic for the subgroup E of type I CRISPR-Cas systems (Makarova et al., 2011). The amino acid sequences of Cas1 and Cas2 are most highly conserved, with >75% identity to related proteins from several other *Alphaproteobacteria*.

Interestingly, the *cas-cse* gene cluser is located adjacent to the CRISPR1 array (5 bp apart). Therefore, these two modules are not separated by an identifiable leader sequence, which in related systems contains external promoters that enable transcription of the CRISPR *locus*.

As shown in Figure 1A, the CRISPR-Cas system is located between two tRNA genes in the CCUG 32053 chromosome (2169951–2184486). The analogous location in the genomes of *P. yeei* strains FDAARGOS_252 and TT13 is occupied by different *loci*: a complete prophage region and an integrase gene of phage origin, respectively.

The CRISPR1 *locus* of CCUG 32053 encompasses 41 spacer sequences, each bordered by an identical 29-bp-long direct repeat (DR) (5′-GGCTCCCCCGCACCCGCGGGGATAGACCC-3′). The CRISPR2 (1251854–1252186) contains only five spacers and its 25-bp-long DRs show significant sequence similarity to those of CRISPR1 (5′-CTGTTCCCCGCATGCGCGGGGATGA-3′; nucleotides common to the DRs of both CRISPRs are underlined). The spacer sequences of both *loci* are unique in the CCUG 32053 genome, and they do not show significant similarity to any known nucleotide sequences of phage origin.

CRISPR *loci* are also present in the strains FDAARGOS_252 and TT13. The former strain contains one set of CRISPR, while the latter has two orphan *loci* not associated with *cas* genes, one of which contains six DRs identical to those of CRISPR1 of CCUG 32053 (Figure 1A).

### Dominant, Species-Specific and Strain-Specific Genes

**FIGURE 2:**
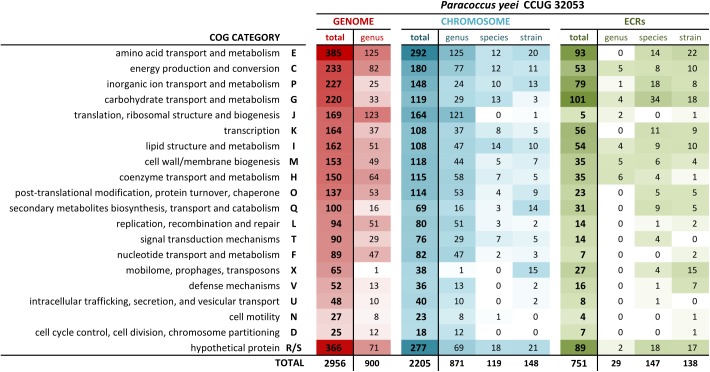
Distribution of COG functional categories within the CCUG 32053 genome, the core genome of the genus *Paracoccus* as well as species- and strain-specific pools of genes.

Functional categorization of the predicted proteins of *P. yeei* CCUG 32053 allowed us to assign COG numbers to approximately 55% of them. The proportion of the proteins in each COG category is shown in Figure 2. Besides poorly characterized or uncharacterized proteins (in the R and S categories), the largest fraction of the classified proteins (13%) was assigned to the group gathering those involved in amino acid transport and metabolism (E category). The next most abundant COGs contain proteins involved in energy production and conversion (C), inorganic ion transport and metabolism (P), and carbohydrate transport and metabolism (G) (Figure 2). Proteins in the aforementioned four groups constitute 36% of the CCUG 32053 proteome.

In the core genome of the genus *Paracoccus* (i.e., genes conserved in all completely sequenced strains), COG category E constitutes the largest gene group, while genes encoding proteins involved in carbohydrate (G) and inorganic ion transport and metabolism (P) occur much less frequently. This indicates that the two latter classes of genes may be responsible for specific adaptations of a strain to their particular ecological niche. Many of these genes are located on ECRs (approximately 50 and 33% in the case of categories P and G, respectively), which suggests their horizontal transmission (Figures 1B, 2).

Interestingly, more than 50% of genes considered species-specific (i.e., present in all *P. yeei* strains but absent in other *Paracoccus* spp.) are also carried by ECRs (Figures 1B, 2). They include genes putatively involved in the utilization of different carbon compounds and energy sources (carbon metabolism and transport of carbon compounds) as well as transporters of inorganic compounds, and genes involved in other processes such as energy metabolism (e.g., putative cytochrome c oxidase) or protein folding (e.g., putative heat shock proteins) (Supplementary Table S5).

The functional composition of genes specific to *P. yeei* CCUG 32053 (i.e., unique in the entire genus) is similar to that of the species-specific set of genes, with potentially adaptive genes involved in transport and metabolism of various carbon compounds forming a dominant group. However, this pool of genetic information contains a noticeably larger proportion of genes characteristic for mobile elements (Figure 2).

As highlighted above, transporter genes constitute a considerable part of the CCUG 32053 genome (448 genes listed in Supplementary Table S6), with members of the ABC family (ATP-binding cassettes) being especially prevalent (Wilkens, 2015) (295 genes). This is the most abundant functional group of genes in the CCUG 32053 genome, and they are predicted to be involved in the transport of various molecules including amino acids, inorganic ions, carbohydrates, and molecules connected with resistance or virulence, i.e., cofactors, lipids or organic ions (Supplementary Table S6).

Transporter genes are also very common in ECRs. Most are located within the two largest replicons, pYEE2 (65 CDSs, 53 of which are of the ABC type) and pYEE1 (55 CDSs, including 40 ABC type), while a lesser number are present in pYEE3 and pYEE4 (Supplementary Table S6). In all cases these transporters are involved in the transfer of basic molecules – mostly amino acids, carbohydrates and inorganic ions.

Tripartite ATP-independent periplasmic (TRAP) transporters (Mulligan et al., 2011) are less abundant in strain CCUG 32053. Genes encoding TRAP transporters were identified in the chromosome (26) and within the 3 largest ECRs (Supplementary Table S6). The latter are mainly involved in amino acid and carbohydrate transport (pYEE1), inorganic ion influx (pYEE2) and the transport of unknown substrates (all in pYEE3). TRAP transporters for all of the aforementioned substrate categories are also encoded by chromosomal genes (Supplementary Table S6).

A third category of transporters – proteins of the major facilitator superfamily (MFS) – comprises mainly multiple-substrate transporters. These enable the transfer of small compounds, such as sugar phosphates, nucleosides, amino acids and small peptides, across the cell membrane (Quistgaard et al., 2016). These proteins act mainly as uniporters or co-transporters. In the CCUG 32053 genome they are encoded within both the chromosome (21) and ECRs (5) (Supplementary Table S6).

### Metabolic Potential of *P. yeei* CCUG 32053

Following the functional assignment of proteins the metabolic potential of strain CCUG 32053 was investigated. To that end, the metabolic pathway profiles of the studied strain and the other 6 sequenced *Paracoccus* spp. were compared using tools available at the KEGG database.

*Paracoccus yeei* CCUG 32053 resembles many other paracocci in being capable of methylotrophy, i.e., the utilization of C1 compounds as sole carbon and energy sources (Baker et al., 1998). The studied strain can be classified as an autotrophic methylotroph due to the presence of genes for proteins involved in the Calvin cycle and the absence of serine cycle enzymes (Dziewit et al., 2015a). Based on comparative analyses, strain CCUG 32053 is predicted to oxidize C1 compounds such as methanol and mono-, di-, and trimethylamine. With regard to carbohydrate metabolism, *P. yeei* CCUG 32053 is presumably able to degrade D-galactonate, D-galacturonate, and D-glucuronate, in contrast to the other analyzed *Paracoccus* species.

Similarly to *P. contaminans* RKI16-01929^T^ and *P. aminophilus* JCM 7686, strains of *P. yeei* lack genes for the sulfur oxidation complex, and therefore are unable to utilize thiosulfate or sulfite as the sole source of energy, unlike many other paracocci (Baker et al., 1998).

Regarding the utilization of electron acceptors other than oxygen, comparative analysis indicates that strain CCUG 32053 is capable of dissimilatory nitrate reduction (respiratory ammonification) via the reactions catalyzed by the NarGHI and NirDB proteins. However, in spite of the presence of gene clusters involved in the reduction of nitric oxide (*nor*) and nitrous oxide (*nos*), no gene for nitrite reductase (*nirS/nirK*) could be identified in the genome. This suggests that the apparent denitrification phenotype of the studied strain must be due to some other process, e.g., conversion of nitrite to nitric oxide in the presence of Fe(II). Such a phenomenon was recently described for *Anaeromyxobacter dehalogenans* and may not be uncommon among bacteria (Onley et al., 2017). The dissimilatory reduction of Fe(III) to Fe(II) – an important step in the proposed scenario – is probably dependent on the activity of PY_02926, a homolog of ferric reductase FerA of *P. denitrificans* PD1222 (Sedlacek et al., 2016).

The genome of *P. yeei* CCUG 32053 was also searched for genes involved in secondary metabolite biosynthesis using the antiSMASH database (Weber et al., 2015). Interestingly, this analysis revealed that pYEE1 carries a complete set of genes for carotenoid synthesis (*crtXYIBZE-idi*, 353406–360835 bp). However, judging by the pale color of its colonies the strain does not produce carotenoids under laboratory conditions. Homologous *crt* gene clusters are also present in extrachromosomal replicons of the two other sequenced *P. yeei* strains: in plasmid 5 of strain FDAARGOS_252 and pTT13-4 of strain TT13.

### Putative Virulence Determinants of *P. yeei* CCUG 32053

Potential virulence determinants of the studied strain were identified *in silico* based on homology to known microbial virulence factors. All proteins encoded by CCUG 32053 and the six other sequenced *Paracoccus* spp. strains were used in BLASTp searches against protein datasets extracted from VFDB and MvirDB. The CCUG 32053 proteins chosen for further inspection were those similar to known virulence factors with close homologs present in the other *P. yeei* strains (one or both), but not in the four other analyzed *Paracoccus* spp. strains. The resultant list of putative virulence-associated proteins for which specific functions could be assigned is shown in Table 2.

**Table 2:** Putative virulence determinants of *P. yeei* CCUG 32053.

#	Gene location	Locus tag (PY32053_)	Predicted function	Closest homologs encoded in complete *P. yeei* genomes	
				*P. yeei* TT13	*P. yeei* FDAARGOS_252
1	chr (GI_Pye_2)	00015	Peptide-methionine (S)-S-oxide reductase (MsrA1)	ATQ55559 [179/179(100%)]	–
		00016	Peptide-methionine (R)-S-oxide reductase (MsrB)	ATQ55560 [147/147(100%)]	ARC36051 [55/116(55%)]
		00019	Peptide-methionine (S)-S-oxide reductase (MsrA2)	ATQ57572 [176/177(99%)]	–
2	chr (GI_Pye_2)	00072	Diguanylate cyclase	ATQ55604 [617/617(100%)]	–
3	chr	00730	Superoxide dismutase	ATQ56160 [220/229(96%)]	ARC37539 [218/229(95%)]
4	chr	00902	Sugar transferase	ATQ56312 [236/238(99%)]	ARC37638 [236/238(99%)]
5	pYEE2	04123	Urease subunit alpha (UreA)	ATQ58125 [565/567(99%)]	ARC34985 [562/567(99%)]
		04124	Urease subunit beta (UreB)	ATQ58124 [101/101(100%)]	ARC34984 [101/101(100%)]
		04125	Urease subunit gamma (UreC)	ATQ58123 [99/100(99%)]	ARC34983 [100/100(100%)]
		04126	Urease accessory protein UreD	ATQ58122 [292/300(97%)]	*^a^*
		04127	Urease accessory protein UreE	ATQ58121 [163/169(96%)]	ARC34982 [165/169(98%)]
		04128	Urease accessory protein UreG	ATQ58120 [216/216(100%)]	ARC34981 [216/216(100%)]
		04129	Urease accessory protein UreF	ATQ58119 [225/231(97%)]	ARC34980 [225/231(97%)]
6	pYEE5	04667	VirD4-like T4SS protein	–	ARC38902 [660/675(98%)]
		04668	VirB11-like T4SS protein	–	ARC38903 [321/329(98%)]
		04669	VirB10-like T4SS protein	–	ARC38904 [411/433(95%)]
		04670	VirB9-like T4SS protein	–	ARC38905 [231/236(98%)]
		04671	VirB8-like T4SS protein	–	ARC38906 [225/225(100%)]
		04672	VirB6-like T4SS protein	–	ARC38907 [348/348(100%)]
		04674	VirB5-like T4SS protein	–	ARC38909 [259/259(100%)]
		04675	Lytic murein transglycosylase	–	ARC38910 [373/377(99%)]
		04677	VirB4-like T4SS protein	–	ARC38911 [781/784(99%)]
		04678	VirB3-like T4SS protein	–	ARC38912 [203/225(90%)]
		04679	VirB2-like T4SS protein	–	*^b^*
		04680	Lytic murein transglycosylase	–	ARC38942 [134/137(98%)]

*^a^The gene for a homolog of the CCUG 32053 and TT13 UreD proteins is frameshifted in the FDAARGOS_252 genome. ^b^The gene for a homolog of the CCUG 32053 VirB2-like protein is present but not annotated in the FDAARGOS_252 genome. The numbers given in the first column refer to the position of the genes for the listed proteins in the CCUG 32053 genome, as shown in Figure 1A. GenBank accession numbers and level of identity are given for the closest homologs of the predicted virulence determinants encoded in the two other complete genomes of P. yeei.*

Intriguingly, using this approach only a limited number of potential virulence determinants present in all three sequenced *P. yeei* genomes could be identified. The most notable is a cluster of genes for urease synthesis located in pYEE2, whose homologs are carried by pTT13-2 of strain TT13 and plasmid 2 of strain FDAARGOS_252. This cluster includes genes for α, β, and γ subunits of urease (UreA, UreB and UreC) as well as four urease accessory proteins (UreD, UreE, UreF, and UreG). Ureases are known to play a role in the survival of pathogenic bacteria in the host and in causing host cell damage and inflammation (Konieczna et al., 2012; Rutherford, 2014). Urease accessory proteins facilitate the maturation of urease by the insertion of two nickel ions at its active site (Fong et al., 2013). Accordingly, genes for nickel ion transport were found in the immediate vicinity of the urease synthesis gene clusters in all three *P. yeei* genomes (data not shown).

Interestingly, while the closest homologs of the identified proteins are found mainly in *Proteobacteria*, the urease subunit γ of CCUG 32053 also shares a high degree of similarity (85–90%) with the corresponding protein of *Cyanobacteria* (e.g., *Anabaena* sp., *Nostoc* sp., and *Scytonema* sp.). Genes encoding urease subunits and accessory proteins are also found on chromosome 1 of *P. denitrificans* PD1222. However, they comprise a gene cluster with a different structure localized within a putative genomic island (as predicted by IslandViewer 4; data not shown) and their products show only moderate levels of similarity to the CCUG 32053 proteins (61–70% for UreABC and 27–65% for UreDEFG). These observations indicate the independent acquisition of the identified gene cluster by *P. yeei* via HGT.

Other putative virulence factors common to the three *P. yeei* strains are chromosomally encoded. These include superoxide dismutases that may participate in the evasion of host defense mechanisms that utilize reactive oxygen species (Miller, 2012; Rigby and DeLeo, 2012). In addition, putative sugar transferases, which exhibit moderate similarity (ca. 50%) to undecaprenyl-phosphate galactose phosphotransferases of *Haemophilus* spp., are probably involved in lipopolysaccharide biosynthesis (Young et al., 2013).

Some of the potential virulence-associated determinants are not shared by all analyzed *P. yeei* strains. Genes for peptide-methionine (S)-S- and (R)-S-oxide reductases (MsrA1, MsrA2 and MsrB) were identified in the chromosomes of strains CCUG 32053 and TT13. Like superoxide dismutases, this class of enzymes is also involved in bacterial defense against the deleterious effects of oxidative stress (Weissbach et al., 2002), and they have been shown to be essential for the virulence of pathogens such as *Salmonella enterica* serovar Typhimurium, *Pseudomonas aeruginosa*, and *Staphylococcus aureus* (Denkel et al., 2011; Romsang et al., 2013; Singh et al., 2015). *P. yeei* CCUG 32053 and TT13 also share a gene encoding a putative protein containing tandem GGDEF and EAL domains, characteristic of hybrid diguanylate cyclases (Seshasayee et al., 2010) involved in the metabolism of cyclic diguanylate. This nucleotide-based second messenger has numerous biological roles including the regulation of virulence gene expression (Tamayo et al., 2007).

Finally, clusters of genes encoding the elements of a putative type IV secretion system (T4SS) were identified in pYEE5 of strain CCUG 32053 and plasmid 4 of strain FDAARGOS_252, with no apparent homologs in the TT13 genome. These clusters contain genes for all archetypical T4SS components except the VirB7- and VirB1-like proteins. However, two lytic murein transglycosylases encoded within both clusters can fulfill the role of the VirB1 protein in the local lysis of the peptidoglycan cell wall that facilitates T4SS assembly (Chandran Darbari and Waksman, 2015). In pathogenic bacteria, T4SSs drive the translocation of effector proteins into eukaryotic target cells, and thus play a prominent role in infection (Gonzalez-Rivera et al., 2016).

### Comparative Genomics of *P. yeei* Strains

Multiple alignment of the three complete *P. yeei* genomes (Figure 1A) demonstrates a relatively high degree of synteny between their chromosomal sequences, especially for strains CCUG 32053 and TT13. The lack of any major sequence rearrangements between the chromosomes and ECRs (except multi-copy ISs) is noteworthy. As shown in Figure 1B, the correspondence among ECRs is less well conserved, with significant reshuffling that clearly distinguishes CCUG 32053 from the two other strains. In particular, while the sequence content of plasmids pTT13-1, pTT13-5, and pTT13-4 of strain TT13 is highly similar to that of plasmids 1, 3, and 5 of strain FDAARGOS_252, respectively, the homologous genetic information in strain CCUG 32053 is distributed between replicons pYEE1 and pYEE4 (Figure 1B). Moreover, some ECRs, namely pYEE3, pTT13-3 and plasmids 4 and 7 of strain FDAARGOS_252, constitute apparent ‘hot spots’ for the integration of TEs. Similar IS-rich regions are also found in the variable regions of the chromosomes (Figure 1A).

It is well known that ISs can mediate large-scale genomic rearrangements (Lee et al., 2016; Lasek et al., 2017). Comparative analysis of the sequenced *P. yeei* genomes enabled us to predict only one occurrence of this phenomenon: an inversion of a 217-kb-long segment of the chromosomal DNA in strain FDAARGOS_252 (1406222–1623414) (Figure 1A). In comparison to the corresponding region of the CCUG 32053 chromosome (2842620–3049478), the inverted DNA region in FDAARGOS_252 is flanked by two copies of IS*Pye28* (IS*481* family) placed in opposite orientations, which could lead to homologous recombination.

Close inspection of the variable regions of the three genomes allowed us to identify two putative integrative elements in the chromosome of strain CCUG 32053, provisionally designated as genomic islands (GIs): GI_Pye_1 and GI_Pye_2 (3064467–3172084 and 3414541–83036, respectively) (Figure 1A). Highly homologous regions were found in the genomes of *P. yeei* TT13 (GI_Pye_2a; Figure 1A) distantly phylogenetically related *Alphaproteobacteria* (e.g., *Pannonibacter* sp., *Brevundimonas* sp., *Methylobacterium* sp.) but not in other *Paracoccus* spp. This strongly suggests that these are (or used to be) true mobile elements capable of horizontal transmission. Although the CCUG 32053 GIs differ in size and structure, they share some common features. Both encode (i) serine or tyrosine family recombinases, (ii) components of plasmid conjugation machinery, (iii) putative REP_3 domain-containing proteins related to plasmid replication initiators, and (iv) putative plasmid partitioning proteins. These observations suggest that the GIs originate from (or contain) integrated plasmids. Analogous GIs are also present in the genomes of strains FDAARGOS_252 and TT13. These are different elements but they fulfill the first three of the criteria listed above. Strain FDAARGOS_252 contains two such elements, designated by us GI_Pye_3 (approximately 1643831–1817825) and GI_Pye_4 (approximately 154120–231318). Strain TT13 contains GI_Pye_2a (1363287–1451351) and GI_Pye_5 (3058627–3090399) (Figure 1A). Similarly to the prophage-related sequences, these GIs tend to be flanked by tRNA genes (Figure 1A) (Williams, 2002).

## Discussion

The results of this study provide the first deep insight into the genomic structure and composition of *P. yeei* – the only species of the genus *Paracoccus* associated with opportunistic human infections. The analysis revealed that *P. yeei* CCUG 32053 – a strain isolated in the United States from a patient with an eye infection – has a composite genome containing eight ECRs of diverse structure and properties. Interestingly, the ECRs carry more than 50% of the genes considered to be specific for *P. yeei* (i.e., not present in other *Paracoccus* spp.; Table 1 and Figure 2), which points to a significant role for these replicons in the evolution of this species.

**FIGURE 3:**
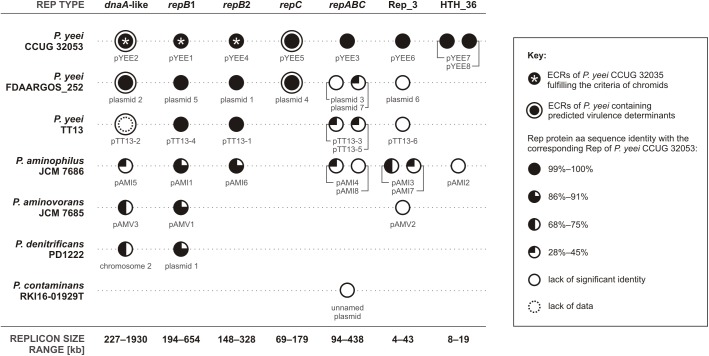
Distribution of conserved REP regions within ECRs of complete *Paracoccus* spp. genomes. [The sequence of pTT13-2 of *P. yeei* strain TT13 (a *dnaA*-like replicon type in this figure) (acc. no. NZ_CP024424) contains neither a gene encoding a DnaA-like initiator nor genes encoding any other recognizable Rep proteins, which suggests that the sequence might be incomplete. Based on the high sequence similarity and conservation of gene synteny with *dnaA*-like replicons of the two other *P. yeei* strains as well as the presence of a PAR region characteristic of *dnaA*-like replicons, pTT13-2 has been included in the *dnaA*-like replicon group for this comparative analysis].

**FIGURE 4:**
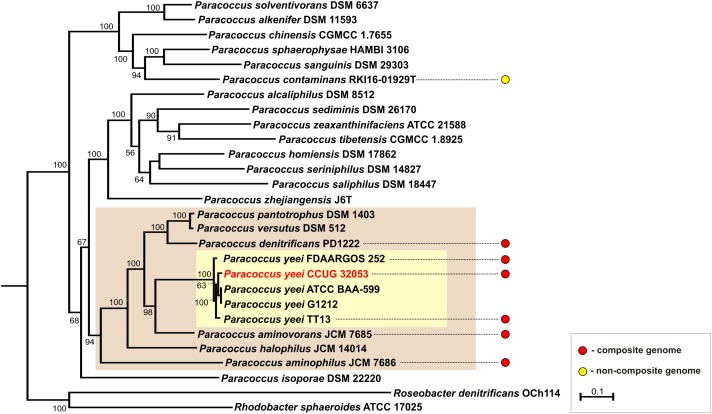
Phylogenetic tree of the genus *Paracoccus* based on concatenated nucleotide alignment of seven core genes (*atpD*, *dnaA*, *dnaK*, *gyrB*, *recA*, *rpoB*, and *thrC*). Homologous genes of *Rhodobacter denitrificans* OCh 114 and *Roseobacter sphaeroides* ATCC 17025 were used as an outgroup. The tree was constructed by applying the Maximum Likelihood algorithm. Statistical support for the internal nodes was determined by 2000 bootstrap replicates and values of ≥50% are shown. The scale bar represents 0.1 substitutions per nucleotide position. The clades of *Paracoccus* spp. strains containing multipartite genomes and of *P. yeei* strains were shown on beige and yellow background, respectively.

To date 7 complete genomic sequences of *Paracoccus* spp. have been deposited in the GenBank database. All but one (*P. contaminans* RKI16-01929^T^) have multipartite genomes, which include 36 ECRs in total (Figure 3). As shown in Figure 4 these strains are localized in one cluster on the phylogenetic tree of the genus *Paracoccus*. The REP regions of their ECRs are widely distributed among plasmids of other *Alphaproteobacteria* (Petersen et al., 2013). As confirmed in the present study, a common feature of these ECRs is their relatively narrow host range, which may restrict the horizontal transmission of exogenous DNA between different classes of *Proteobacteria*.

The multireplicon genome architecture of *Paracoccus* spp. was previously analyzed in *P. denitrificans* PD1222 (type strain of the genus), *P. aminophilus* JCM 7686 (Dziewit et al., 2014) and *P. aminovorans* JCM 7685 (Czarnecki et al., 2017). Besides a chromosome and dispensable plasmids, each of these strains contains a chromid – an essential ECR of plasmid origin carrying a set of housekeeping genes. The essential nature of the chromids of *P. aminophilus* JCM 7686 and *P. aminovorans* JCM 7685 was verified experimentally by a target-oriented curing technique, based on the incompatibility phenomenon (Dziewit et al., 2014; Czarnecki et al., 2017). Unfortunately, such a strategy failed in the case of *P. yeei* CCUG 32053, since the majority of ECRs of this strain contain toxin-antitoxin systems that confer their apparent “essentiality” (Czarnecki et al., 2015). Chromids of this strain were therefore distinguished *in silico* by verifying a set of core criteria (Harrison et al., 2010). This analysis revealed that three large ECRs of strain CCUG 32053 (pYEE1, pYEE2, and pYEE4) (i) carry plasmid-type REP regions, (ii) have a nucleotide composition that is close to that of the chromosome (less than 1% difference in G + C content), and (iii) possess a set of core genes (in one copy in the genome) characteristic for the entire genus (Table 1). The presence of core genes within ECRs is the result of inter-replicon transfer of chromosomal genes into co-residing plasmids, and indicates the long co-evolution of these replicons with the chromosome, which has led to the essentiality and evolutionary conservation of chromids.

The core genes located within the CCUG 32053 chromids are involved in diverse metabolic processes, which may be essential for their host strain. The largest chromid, pYEE1, carries genes possibly required for (i) biosynthesis of the lipoyl cofactor, necessary for the function of several key enzymes involved in oxidative metabolism (lipoyl synthetase LipA; PY_03888), (ii) the breakdown of leucine (methylcrotonyl-CoA carboxylase biotin-containing and carboxyl transferase subunits; PY_03816 and PY_3817), and (iii) electron transport via the respiratory chain (complex I) (PY_03686). Another chromid, pYEE2, encodes (i) acetyl-CoA *C*-acyltransferase (PY_04023) – an enzyme participating in several important metabolic pathways, (ii) proteins involved in cobalamin biosynthesis (PY_04096, PY_04098, PY_04099), (iii) proteins for the transport and utilization of glycerol (PY_04083–04091), and (iv) a putative ABC transporter ATP-binding protein (PY_04004). The core genes of the smallest chromid, pYEE4, encode the complete pathway for the synthesis of thymidine diphosphate (TDP)-l-rhamnose (PY_04496–04499), an important substrate in the biosynthesis of lipopolysaccharides (LPS) and in modification of translation elongation factor P (EF-P), which are common processes affecting the level of virulence of many pathogenic Gram-negative strains (Tsukioka et al., 1997; Giraud et al., 2000; Lassak et al., 2015). The EF-P itself (PY_04509), which is essential for cell viability (Aoki et al., 1997), as well as an enzyme modifying this factor (EF-P-lysine aminoacylase l, PY_04508) (Yanagisawa et al., 2010), are also encoded within pYEE4. It is striking that all of the aforementioned core genes are also localized extrachromosomally within predicted chromids of the two other sequenced *P. yeei* strains, FDAARGOS_252 and TT13. As shown in Figure 1B, the chromid-encoded pool of genes is conserved in *P. yeei* (although partially shuffled between different ECRs), which indicates that these replicons contribute significantly to the species-specific properties of these bacteria.

Two types of replicons that are characteristic for all multipartite genomes of *Paracoccus* spp. were distinguished by comparative analysis. These large replicons contain *dnaA*-like or *repB1* REP regions (Figure 3). The former group gathers solely essential chromids, with chromosome 2 of *P. denitrificans* PD1222 being the first replicon of this type identified in the genus *Paracoccus*. The latter group (with the most highly conserved REP sequences; Figure 3) includes lifestyle-determining ECRs of *P. aminophilus* JCM 7686 and *P. aminovorans* JCM 7685, which provide versatile niche-specific adaptations to their host strains (Dziewit et al., 2014; Czarnecki et al., 2017). In the case of *P. yeei* CCUG 32053, both *dnaA*-like and *repB1* replicons (pYEE2 and pYEE1, respectively; Figure 3) as well as *repB2*-type pYEE4 were classified as chromids, which points to the diversity of inter-replicon recombinational rearrangements in this species. None of the sequenced *P. yeei* strains carry any species-specific replicons, although two of them (CCUG 32053 and FDAARGOS_252) contain *repC*-type REP regions that are unique among the genomes of *Paracoccus* spp. analyzed so far (Figure 3). These replicons do not have a conserved structure and have been significantly shaped by transposition events (Figure 1).

The flexible genome of *P. yeei* consists not only of ECRs; it also has numerous genetic elements integrated within the chromosome. We identified and classified TEs as well as putative genomic islands carried by strains CCUG 32053, FDAARGOS_252 and TT13 (Figure 1A). In total, the genomes of the three strains contain 278 complete and 206 partial ISs, representing 15 IS families. The presence of multiple IS copies may lead to the formation of composite transposons; however, no such element was identified by *in silico* sequence analysis. An intriguing observation is that all but one of the identified ISs represent novel elements. Only one complete element was common to the three strains of *P. yeei*, which suggests that the vast majority of ISs were horizontally acquired independently by the individual strains. The ISs are unevenly distributed within the genomes and their accumulation was apparent at a few locations corresponding to chromosomal regions of exogenous origin or particular ECRs (Figure 1A).

The availability of three complete genomes of *P. yeei* allowed us to not only comparatively analyze their structure, but also to attempt to identify putative virulence factors that determine the ability of this species to cause opportunistic infections in humans. *In silico* analysis detected only a handful of potential virulence-associated genes without close homologs in the genomes of other *Paracoccus* species. Their predicted functions – ureolytic activity, effector translocation, oxidative stress response, global regulation of gene expression etc. – are associated with rather non-specific virulence mechanisms. Therefore, without further experimental evidence it is difficult to establish which of them (if any) represent critical pathoadaptive traits of this species.

The majority of predicted virulence-associated genes are carried within GIs and ECRs, and therefore their presence is likely to be the result of horizontal transmission. Besides the acquisition of exogenous genetic determinants, the re-appropriation of preexisting non-pathoadaptive paracoccal traits is likely to have been decisive in the evolution of *P. yeei* virulence. A similar scenario of gene cooption – i.e., modification of the expression and function of genes not originally associated with virulence – has been proposed to explain the evolution of pathogenicity in other bacterial species, including rhodococci and mycobacteria (Letek et al., 2010; Singh et al., 2014). The preliminary analysis performed in this study was focused primarily on the identification of homologs of known virulence factors, so novel genetic determinants conferring a pathogenic lifestyle may be uncovered in the future.

## Author Contributions

RL performed the comparative genomic analyses, predicted the *in silico* metabolic potential of *P. yeei*, and deposited genomic sequence in the database. RL and MS assembled the genomic sequence and identified putative virulence factors. MS and MM manually annotated the sequence, constructed shuttle plasmids, and analyzed plasmid host range and physiological properties of CCUG 32053. CC, MS, and DB identified and characterized TEs. PD performed the genome-scale comparative bioinformatic analyses, identified and described prophage regions, and performed phylogenetic analysis. DS, AP, and JC analyzed genomic data. DB obtained funding, designed the study, and analyzed the data. DB and RL wrote the final version of the manuscript. All authors approved the manuscript for publication.

## Conflict of Interest Statement

The authors declare that the research was conducted in the absence of any commercial or financial relationships that could be construed as a potential conflict of interest.

## Footnotes

1 https://github.com/rrwick/Porechop

2 http://www.ncbi.nlm.nih.gov/

## Supplementary Material

FIGURE S1 Extrachromosomal replicons of *Paracoccus yeei* CCUG 32053 visualized by electrophoretic methods **(A)** and circular representations of the *P. yeei* CCUG 32053 genome **(B)**. Circles display (from the outside): (i) predicted CDSs transcribed in the clockwise direction, (ii) predicted CDSs transcribed in the counterclockwise first direction, (iii) the GC percent deviation, (iv) GC skew. The circles are not drawn to scale.

TABLE S1 Plasmids and oligonucleotide primers used in this study.

TABLE S2 Detailed description of comparative genomic analyses performed using EasyFig and Circoletto.

TABLE S3 Minimal inhibitory concentration (MIC) of selected antimicrobial compounds against *P. yeei* CCUG 32053.

TABLE S4 Insertion sequences identified *in silico* in the genome of *P. yeei* CCUG 32053.

TABLE S5 Species- and strain-specific genes of *P. yeei* CCUG 32053.

TABLE S6 Transporter genes identified *in silico* in the genome of *P. yeei* CCUG 32053.
