# Supplementary material for: Genome Structure of the Opportunistic Pathogen Paracoccus yeei (Alphaproteobacteria) and Identification of Putative Virulence Factors
Source: Front Microbiol. 2018 Oct 25;9:2553. doi: 10.3389/fmicb.2018.02553 (PMC6209633; doi:10.3389/fmicb.2018.02553)
Supplement: FIGURE S1 — Extrachromosomal replicons of Paracoccus yeei CCUG 32053 visualized by electrophoretic methods (A) and circular representations of the P. yeei CCUG 32053 genome (B). Circles display (from the outside): (i) predicted CDSs transcribed in the clockwise direction, (ii) predicted CDSs transcribed in the counterclockwise first direction, (iii) the GC percent deviation, (iv) GC skew. The circles are not drawn to scale. [file Image_1.PDF]

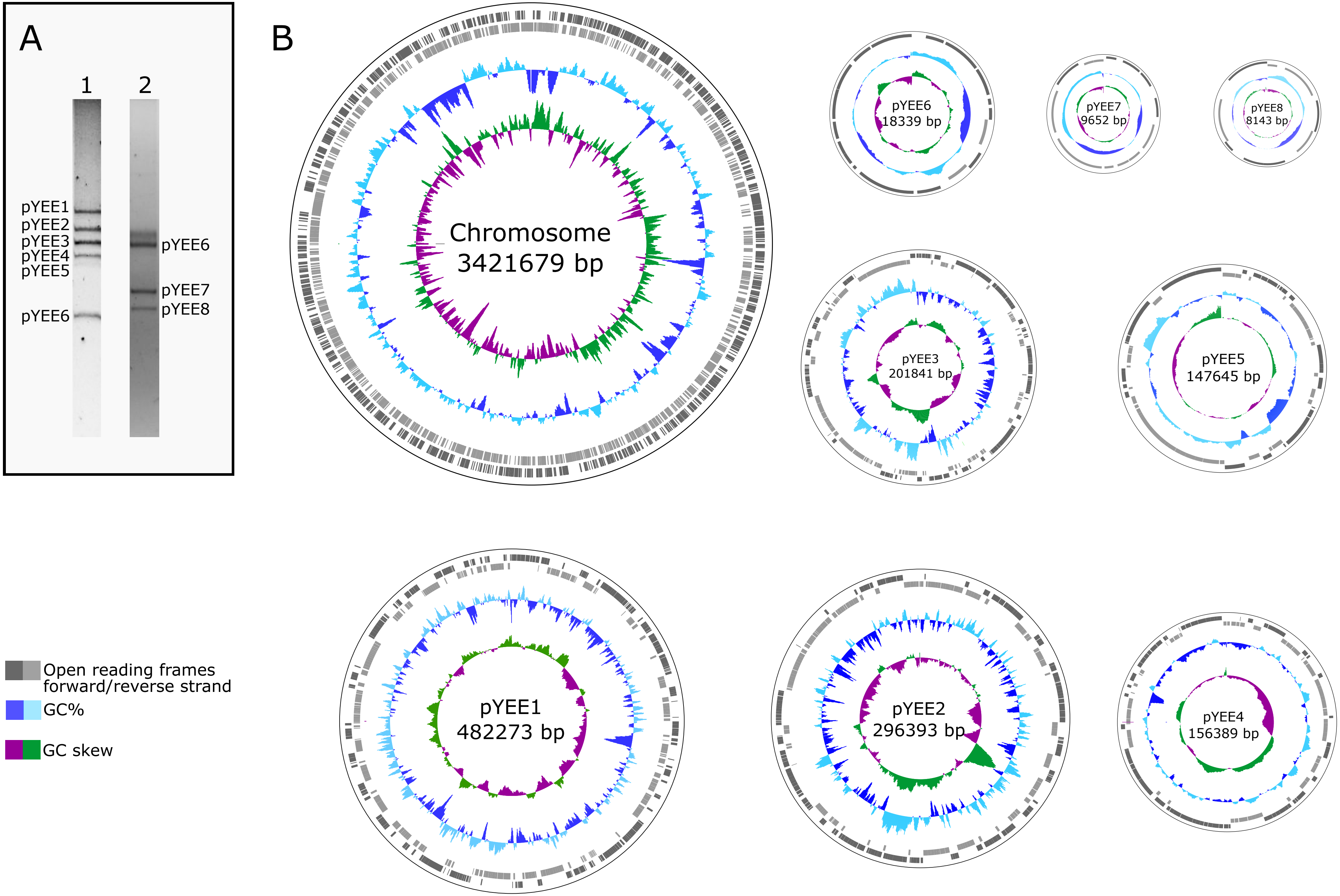

FigS1
Extrachromosomal replicons of P. yeei CCUG 32053 visualized by electrophoretic methods (A) and circular representations of the P. yeei CCUG 32053 genome (B).
Circles display (from the outside): (i) predicted CDSs transcribed in the clockwise direction, (ii) predicted CDSs transcribed in the counterclockwise direction, (iii) the GC percent deviation, (iv) GC skew (G-C/G+C). Circles are drawn not in scale. Generated using DNAPlotter.
