# Supplementary material for: Genome Structure of the Opportunistic Pathogen Paracoccus yeei (Alphaproteobacteria) and Identification of Putative Virulence Factors
Source: Front Microbiol. 2018 Oct 25;9:2553. doi: 10.3389/fmicb.2018.02553 (PMC6209633; doi:10.3389/fmicb.2018.02553)
Supplement: TABLE S1 — Plasmids and oligonucleotide primers used in this study. [file Table_1.DOC]

**TABLE S1.** Plasmids and oligonucleotide primers used in this study.

| **Plasmid** | **Description** | **Source or reference** |
| --- | --- | --- |
| pABW1 | Mobilizable cloning vector; Km^r^; *ori* pMB1; *oriT* RK2; *lacZα*; MCS | Bartosik et al., 1997 |
| pABW-YEE1 | Km^r^; ori pMB1; ori pYEE1; oriT RK2  Mobilizable E. coli-Paracoccus spp. shuttle plasmid constructed by insertion of an SacI/XbaI restriction fragment of pYEE1 (containing the plasmid replication system) into the SacI/XbaI site of pABW1. | This study |
| pABW-YEE2 | Km^r^; ori pMB1; ori pYEE2; oriT RK2  Mobilizable E. coli-Paracoccus spp. shuttle plasmid constructed by insertion of an KpnI/XbaI restriction fragment of pYEE1 (containing the plasmid replication system) into the KpnI/XbaI site of pABW1. | This study |
| pABW-YEE3 | Km^r^; ori pMB1; ori pYEE1; oriT RK2  Mobilizable E. coli-Paracoccus spp. shuttle plasmid constructed by insertion of an KpnI/XbaI restriction fragment of pYEE3 (containing the plasmid replication system) into the KpnI/XbaI site of pABW1. | This study |
| pABW-YEE5 | Km^r^; ori pMB1; ori pYEE1; oriT RK2  Mobilizable E. coli-Paracoccus spp. shuttle plasmid constructed by insertion of an KpnI/XbaI restriction fragment of pYEE5 (containing the plasmid replication system) into the KpnI/XbaI site of pABW1. | This study |
| pABW-YEE6 | Km^r^; ori pMB1; ori pYEE1; oriT RK2  Mobilizable E. coli-Paracoccus spp. shuttle plasmid constructed by insertion of an KpnI/XbaI restriction fragment of pYEE6 (containing the plasmid replication system) into the KpnI/XbaI site of pABW1. | This study |
| pABW-YEE7 | Km^r^; ori pMB1; ori pYEE1; oriT RK2  Mobilizable E. coli-Paracoccus spp. shuttle plasmid constructed by insertion of an KpnI/XbaI restriction fragment of pYEE7 (containing the plasmid replication system) into the KpnI/XbaI site of pABW1. | This study |
| pABW-YEE8 | Km^r^; ori pMB1; ori pYEE8; oriT RK2  Mobilizable E. coli-Paracoccus spp. shuttle plasmid constructed by insertion of BamHI restriction fragment of pYEE8 (containing the plasmid replication system) into the BamHI site of pABW1. | This study |
| pMEC1 | Entrapment shuttle vector; Km^r^; *ori* ColE1; *ori* pWKS1; *oriT* RK2; *cI-tetA* | Bartosik et al., 2003 |
| **Primer** | **Sequence (5’->3’)** | **Source or reference** |
| *Primer pairs used for the PCR amplification of REP modules^a^* | | |
| REP_YEE1F | 5’-gtgagctcTCCCATGGTCAGGTTACAAG-3’ | This study |
| REP_YEE1R | 5’-cgtctagaGCTGATGCGTCCTCGCTAAG-3’ | This study |
| REP_YEE2F | 5’-gtggtaccCACCACGGGAATGACGGAAC-3’ | This study |
| REP_YEE2R | 5’-cgtctagaAGGCAAAGCGCGGACGAACG-3’ | This study |
| REP_YEE3F | 5’-gtggtaccTCCTGATGCCGACTGCTAAC-3’ | This study |
| REP_YEE3R | 5’-cgtctagaAGCCGCATTGCGTCCTAGCC-3’ | This study |
| REP_YEE5F | 5’-gtggtaccGGCCACTATGTCGGCATAGG-3’ | This study |
| REP_YEE5R | 5’-cgtctagaGGACTACCAAGGCCACTACG-3’ | This study |
| REP_YEE6F | 5’-gtggtaccTGCCACGGTCCGTGATGAAC-3’ | This study |
| REP_YEE6R | 5’-cgtctagaACGAGCGGAACGCAAGGAAG-3’ | This study |
| REP_YEE7F | 5’-gtggtaccGAGCGTTGACTTGCTCTTTG-3’ | This study |
| REP_YEE7R | 5’-cgtctagaTTTGCCTGCTGTCACCCTTC-3’ | This study |
| *Primer pairs used for the PCR amplification of cI-tetA specific regions of entrapment vector pMEC1* | | |
| ALIS | 5’-TTGTAATCAGCTATGCGCCG-3’ | Bartosik et al., 2003 |
| ARIS | 5’-TCTGGCTTGAGGTTGAAGGT-3’ | Bartosik et al., 2003 |
| BLIS | 5’-TGGTGCGGTCATGGAATTAC-3’ | Bartosik et al., 2003 |
| BRIS | 5’-GTATGCAGCCGTCACTTAGA-3’ | Bartosik et al., 2003 |
| CLIS | 5’-TCCCTGCCTGAACATGAGAA-3’ | Bartosik et al., 2003 |
| CRIS | 5’-ACACAAGAGCAGCTTGAGGA-3’ | Bartosik et al., 2003 |
| DLIS | 5’-TCTTGTCTGCGACAGATTCC-3’ | Bartosik et al., 2003 |
| DRIS | 5’-TTCATACACGGTGCCTGACT-3’ | Bartosik et al., 2003 |

^a^ Nucleotides not complementary to the *P. yeei* plasmids sequence are shown in lowercase letters. Introduced KpnI, SacI and XbaI restriction sites are underlined.

|  |  |
| --- | --- |

References

**Bartosik, D., Bialkowska, A., Baj J., and Wlodarczyk, M. (**1997) Construction of mobilizable cloning vectors derived from pBGS18 and their application for analysis of replicator region of a pTAV202 mini-derivative of Paracoccus versutus pTAV1 plasmid. *Acta Microbiol Polon* 46**:** 379-383.

Bartosik, D., Sochacka, M., and Baj, J. (2003b) Identification and characterization of transposable elements of *Paracoccus pantotrophus*. *J Bacteriol* 185: 3753-3763.
