# Supplementary material for: Genome Structure of the Opportunistic Pathogen Paracoccus yeei (Alphaproteobacteria) and Identification of Putative Virulence Factors
Source: Front Microbiol. 2018 Oct 25;9:2553. doi: 10.3389/fmicb.2018.02553 (PMC6209633; doi:10.3389/fmicb.2018.02553)
Supplement: TABLE S2 — Detailed description of comparative genomic analyses performed using EasyFig and Circoletto. [file Table_2.DOC]

**TABLE S2.** Detailed description of comparative genomic analyses performed using EasyFig and Circoletto (presented in Fig. 1).

| **Tool** | **Parameters** | **Arbitrary start of the compared sequences^a^** |
| --- | --- | --- |
| EasyFig | Comparison 1: *P. yeei* TT13 vs *P. yeei* CCUG 32053  Comparison 2: *P. yeei* CCUG 32053 vs *P. yeei* FDAARGOS_252  BLASTn options: ‒ min. identity: 90%  ‒ max. e-value: 1e-40  ‒ min. length: 1000 bp | *P. yeei* CCUG 32053: ‒ chromosome: 1 997 041  *P. yeei* FDAARGOS_252:  ‒ chromosome: 462 346 |
| Circoletto | Query: ECRs of *P. yeei* CCUG 32053  Database: ECRS of *P. yeei* TT13 and FDAARGOS_252  Sequence orientation:  ‒ query: normal  ‒ database: reverse-complement (except plasmid 4 of FDAARGOS_252)  BLASTn options:  ‒ min. identity: 90%  ‒ max. e-value: 1e-40 | *P. yeei* TT13: ‒ pTT13-1: 55 337 ‒ pTT13-2: 92 791 ‒ pTT13-4: 53 441  *P. yeei* FDAARGOS_252: ‒ plasmid 1: 61 747 ‒ plasmid 2: 205 881 |

^a^ Chosen for a greater clarity of presentation. All other sequences start at the coordinate 1 as deposited in the databases
