# Supplementary material for: Genome Structure of the Opportunistic Pathogen Paracoccus yeei (Alphaproteobacteria) and Identification of Putative Virulence Factors
Source: Front Microbiol. 2018 Oct 25;9:2553. doi: 10.3389/fmicb.2018.02553 (PMC6209633; doi:10.3389/fmicb.2018.02553)
Supplement: TABLE S3 — Minimal inhibitory concentration (MIC) of selected antimicrobial compounds against P. yeei CCUG 32053. [file Table_3.DOC]

**TABLE S3.** Minimal inhibitory concentration (MIC) of selected antimicrobials against
*P. yeei* CCUG 32053.

| **Antimicrobial** | **MIC range**  **(μg/ml)** | **MIC**  **(μg/ml)** |
| --- | --- | --- |
| Ampicillin | 0.125-128 | 16 |
| Chloramphenicol | 0.125-128 | 1 |
| Ciprofloxacin | 0.125-128 | 1 |
| Erythromycin | 0.063-128 | 0.5 |
| Kanamycin | 0.125-128 | 4 |
| Spectinomycin | 0.125-128 | 4 |
| Streptomycin | 0.125-128 | 4 |
| Tetracycline | 0.063-128 | 0.125 |
